# 50 years on and still very much alive: ‘Apoptosis: a basic biological phenomenon with wide-ranging implications in tissue kinetics’

**DOI:** 10.1038/s41416-022-02020-0

**Published:** 2022-11-11

**Authors:** Christoph Nössing, Kevin M. Ryan

**Affiliations:** 1 Cancer Research UK Beatson Institute, Glasgow, G61 1BD UK; 2 School of Cancer Sciences, University of Glasgow, Garscube Estate, Switchback Road, Glasgow, G61 1QH UK

**Keywords:** Cell death, Cancer

## Abstract

Cell death is part of the lifecycle of every multicellular organism. Nineteenth-century pathologists already recognised that organised forms of cell death must exist to explain the demise and turnover of cells during metamorphosis (of insects), embryogenesis and normal tissue homoeostasis [1]. Nevertheless, Kerr, Wyllie and Currie in their seminal paper of 1972, were the first to collate and define the distinct morphological features of controlled cell death in different contexts [2]. To describe the processes of cell deletion observed under both physiological and pathological conditions, they coined the term ‘Apoptosis’ (derived from the Greek word ‘ἀπόπτωσις’, meaning ‘dropping off or falling off’ of petals from flowers). Kerr, Wyllie and Currie defined apoptosis as a mechanism ‘*complementary to mitosis in the regulation of animal cell populations’*. In addition, they already recognised the potential to use this programmed form of cell death for cancer therapy, but they also emphasised the occurrence of apoptosis during cancer development. In this article, some 50 years after its initial publication in *The British Journal of Cancer*, we revaluate and put the authors initial assumptions and general concepts about apoptosis into the context of modern-day biology

## Morphology and molecular mechanisms of apoptosis

**Fig. 1:**
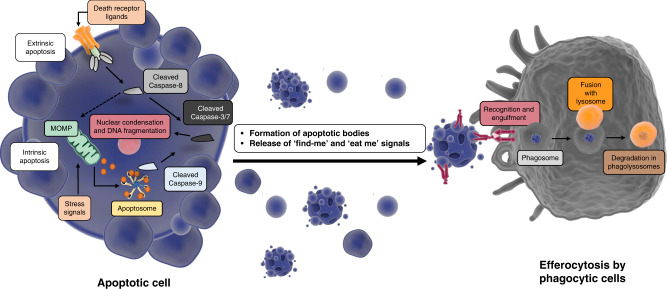
Morphological and molecular events in apoptosis. Despite 50 years of progress on the delineation of the molecular mechanisms of apoptosis, the morphological characteristics of apoptosis as described by Kerr, Wyllie and Currie, including nuclear condensation, membrane blebbing, cell fragmentation, formation of apoptotic bodies and engulfment by phagocytic cells are still very much accepted as cellular events associated with apoptotic death. Figures were generated with the aid of the Library of Science and Medical Illustrations.

Key characteristics of apoptosis, as described by Kerr, Wyllie and Currie, were that it is a two-stage cell death mechanism consisting of the ‘*formation of apoptotic bodies’* (described as ‘*small […], spherical […] cytoplasmic fragments’*) and *‘their phagocytosis and degradation by other cells’*. Apoptotic cells display distinct morphological features, including separation from other cells, nuclear condensation, membrane blebbing, fragmentation and cell shrinkage, and the formation of apoptotic bodies. Released apoptotic bodies are then cleared by cells with phagocytic activity and are degraded in the lysosome (Fig. 1). Importantly, these distinct morphological features of apoptotic bodies are still used nowadays to histologically detect apoptosis and to distinguish it from other forms of cell death.

The molecular and biochemical mechanisms behind apoptosis were completely unknown at the time of the Kerr, Wyllie and Currie publication, but have been unravelled and highly characterised over the past decades (see ref. [3]). Little was also known about the initiating factors of apoptosis, but there was already scientific evidence that ‘*apoptosis can be promoted or inhibited […] by hormone withdrawal or stimulation’*. (e.g., ACTH deprivation causes apoptosis in the adrenal cortex of rats [4]). Furthermore, Kerr and Searle also showed in a separate publication that irradiation of human squamous cell carcinomas can cause widespread apoptosis [5]. It is now well established in the cell death field, that apoptosis can be triggered via two different pathways, depending on the origin of the cell death-inducing perturbations. Intracellular stresses can lead to intrinsic apoptosis, whereas activation of death receptors or dependence receptors stimulates the extrinsic apoptosis pathway [3].

Intrinsic apoptosis is controlled by the B-cell lymphoma 2 protein (BCL-2) family. Several intracellular events (e.g. DNA damage, hypoxia, starvation, cytokine deprivation or high levels of ROS) can cause an imbalance in the BCL-2 protein family, which consists of anti- and pro-apoptotic members. They can be distinguished by their amount of BCL-2 homology domains (BH). Anti-apoptotic proteins like BCL-2, BCL-xL and MCL-1 have the domains BH1-4, while pro-apoptotic proteins like BAX, BAK and BOK have only the BH1-BH3 domains. In healthy cells, anti-apoptotic proteins bind to their pro-apoptotic counterparts and inactivate them. Apoptotic stressors can reduce the amount of anti-apoptotic BCL-2 proteins or stimulate the production and the release of a third member of the BCL-2 family: the BH3-only proteins (i.e., PUMA, NOXA, BIK, BIM, BID, BMF and BAD) [6]. These pro-apoptotic proteins can neutralise the anti-apoptotic effects of BCL-2, BCL-xL and MLC-1 by binding to them. Thereby BAX and BAK (the effector proteins of intrinsic apoptosis [7]) are no longer inhibited, they can homo-oligomerize and insert themselves into to the outer mitochondrial membrane and promote mitochondrial outer membrane permeabilisation (MOMP) [8, 9]. This membrane disruption causes the release of different mitochondrial intermembrane proteins to the cytosol. Among other proteins, cytochrome-*c* is the main activator of apoptosis downstream of MOMP. It recruits apoptotic protease-activating factor-1 (APAF-1) and forms the apoptosome [10]. This complex activates caspase-9 that leads eventually to the activation of executioner caspases and apoptosis.

On the other hand, the extrinsic pathway is triggered by perturbations of the extracellular microenvironment. The starting point of extrinsic apoptosis is the binding of extracellular ligands such as tumour necrosis factor (TNF), TNF-related apoptosis-inducing ligand (TRAIL) or Fas ligand (Fas-L or CD95-L) to the extracellular domain of their corresponding death receptors (i.e., type 1 TNF receptor (TNFR1), DR5 (TRAIL-Receptor) or Fas receptor, respectively). TRAIL or FAS-L binding leads to receptor trimerization and to the intracellular exposure of a death domain (DD), which acts as a binding motif for the Fas-associated death domain (FADD). This adaptor protein recruits procaspase-8 and forms the death-inducing signalling complex (DISC). Once established DISC stimulates caspase-8 dimerisation, catalytic cleavage of procaspase-8, and activation of executioner caspases [11]. In contrast, binding of TNF to TNFR1 acts in most cases as a pro-survival signal via the activation of NF-κB [12]. Instead of FADD, TNF-receptor-associated death domain (TRADD) is initially recruited to TNFR1 and forms together with TNF receptor-associated factor 2 (TRAF2), receptor-interacting protein 1 (RIPK1) and cIAP1 and 2 the TNFR1 complex 1. TRAF2 and cIAPs are responsible for modifying RIPK1 with K63-linked ubiquitin chains, which stimulate a signalling cascade to activate NF-κB. Inhibition of cIAPs or active deubiquitination (through CYLD [13]) of RIPK1 leads to its release into the cytoplasm, where RIPK1, FADD and procaspase-8 form complex IIb, which initiates apoptosis [14].

The intrinsic and extrinsic pathways, under certain circumstances, can influence each other to induce apoptosis. Active caspase-8 can cleave the pro-apoptotic protein BID at the N-terminus to form a truncated form of BID (tBID) [15]. tBID then translocates to mitochondrial membranes, binds to BAX and facilitates the insertion of BAX in the mitochondrial outer membrane, culminating in MOMP. BCL-xL inhibits tBID binding to BAX. BAD in return is responsible for releasing this block [16]. Another connecting player is SMAC. Like cytochrome-*c*, it is released from the mitochondrial intermembrane space after MOMP. SMAC can disrupt the inhibitory effect of XIAP [17] on caspases and thereby promote apoptosis.

Both extrinsic and intrinsic apoptosis pathways culminate in the activation of executioners, such as caspases-3. These cysteine-dependent aspartate-directed proteases (caspases), cleave hundreds of target proteins to trigger apoptotic cell death [18]. For example, the cleavage of Acinus [19] and iCAD (inhibitor of caspase-activated DNAse) [20] proteins causes chromatin condensation and DNA fragmentation, and cleavage of the ROCK1 kinase causes reorganisation of actin filaments and membrane blebbing [21]. Ultimately, the distinct morphological features of apoptotic cells described by Kerr, Wyllie and Currie are the work of executioner caspases. While several key targets of executioner caspases have been identified, inhibiting the cleavage of one component is not sufficient to block apoptosis. Therefore the activation of executioner caspases is the key event that causes death in apoptotic cells [22].

Apoptosis is a molecular process that plays a major role during development and tissue homoeostasis (see below). The constant demise of millions of cells produces vast quantities of apoptotic cells that need to be removed to maintain normal tissue architecture and function. Kerr, Wyllie and Currie described that condensed and fragmented apoptotic bodies are phagocytosed by ‘*histiocytes*’ or by surrounding epithelial cells and subsequently degraded intracellularly [2]. This process is nowadays known as efferocytosis [23]. Depending on the tissue, the clearance of apoptotic bodies is performed by a variety of professional, non-professional and specialised phagocytes [24]. In order to attract phagocytes, dying cells release distinct ‘find-me signals’. These signals include modified membrane lipids (lysophosphatidylcholine (LPC) [25] and sphingosine-1-phosphate (S1P) [26]), chemokines (CX3CL1) [27] and nucleotides (ATP and UTP). ATP, for example, is released through pannexin-1 channels in a caspase-depended manner [28]. Extracellular nucleotides attract phagocytes by binding to their P2Y2 receptors [29]. In addition, dying cells display so-called ‘eat-me’ signals on their surface to guide their own degradation by recruited phagocytes and to protect healthy cells in the microenvironment. The existence of these signals was predicted by Kerr, Wyllie and Currie when they speculated that apoptotic bodies are ‘*rapidly phagocytosed […] because of changes in the properties of their surface membranes’*. The best characterised ‘eat-me’ signal is indeed the lipid composition of the plasma membrane. In healthy cells phospatidylserine (PtdSer) resides mainly in the inner leaflet of the plasma membrane, but is exposed extracellularly on apoptotic cells. The production of this ‘eat-me’ signal is also caspase-dependent. The flippase ATP11 maintains low PtdSer-levels in the outer leaflet, but is inactivated by caspase-3 cleavage [30]. Furthermore, the caspase-3-activated scramblase Xkr8 promotes PtdSer exposure on apoptotic cells [31]. PtdSer is recognised by phagocytic cells via multiple PtdSer membrane receptors (e.g., TIM1, TIM4, BAI1, STABILIN 2) [23].

The activation of PtdSer-receptors on phagocytic cells causes the reorganisation of their plasma membrane and their cytoskeleton to engulf and internalise apoptotic bodies. This process is driven by several pathways that ultimately stimulate actin polymerisation and depolarisation to form vesicles that contain apoptotic bodies, called phagosomes [32]. Kerr, Wyllie and Currie commented once again that they were convinced that ‘*autolysis of phagocytosed apoptotic bodies is a result of their inability to maintain chemical homoeostasis within phagosomes’* and that ‘*lysosome enzymes do […] play a vital role in the further degradation of phagocytosed bodies*’. However, not autolysis, but a well-orchestrated maturation pathway ensures that phagosomes fuse eventually with lysosomes, which are responsible for the degradation of the internalised apoptotic bodies. These degraded components can then be recycled and reused by the phagocytic cell. According to Kerr, Wyllie and Currie ‘[Apoptosis] *is economical in terms of re-utilisation of cell components’*. The molecular mechanisms behind these processes have been excessively described elsewhere [23].

## Apoptosis in development and tissue homoeostasis

The ontogenesis of multicellular organisms is a highly complex and tightly regulated process. While the expansion of body mass is achieved by cell proliferation and differentiation, cell death also plays a major role during development by shaping the final form of an adult organism through the removal of cells. The importance of cell death during the embryogenesis of vertebrates has been described systematically already in the early 1950s [33]. Because of the morphology of dying cells during development*,* Kerr, Wyllie and Currie speculated, that ‘*apoptosis plays a vital role in […] the development of the lumina of tubular structures, the fashioning of the limbs, the formation of interdigital clefts and the involution of phylogenetic vestiges’*. It is now widely recognised that the majority of cell death that occurs during development is indeed apoptosis and the molecular mechanisms behind it have been elucidated [34, 35]. The removal of cells to sculpt digits is a classic example of apoptosis during development. Bone morphogenetic proteins (BMPs) are responsible for inducing apoptosis in the interdigital mesenchyme and this cell removal pathway relies on the BH3-only proteins BIM and BMF [36]. The cavitation or the development of hollow structures is another example where cells are degraded by apoptosis. The outer cells produce an unknown pro-death signal that induces apoptosis in the inner cells to form the cavity, while their interaction with the basement membrane stimulates their own survival [37]. Furthermore, apoptosis also occurs during the selection and demise of excess neurons in the developing sympathetic nervous system in mammals [34]. Impairment of apoptotic mediators leads in most cases to embryonic lethality, further highlighting the importance of a functioning apoptotic machinery during the development of organisms [38].

The mammalian immune system consists of highly specialised cell types and their homoeostasis is widely controlled by apoptosis. Neutrophils, for example, have a very short lifespan of only 5.4 days [39] and it is estimated that 20% these cells die via apoptosis each day in adult humans. This high turnover can be explained by the important role of neutrophils in the phagocytosis of invading pathogens and their subsequent self-destruction [40]. In addition, cells of the adaptive immune system need to be tightly controlled to avoid reacting to self-antigens, which would cause autoimmune diseases. B cells mature in the bone marrow, where the immunoglobulin part of B-cell receptors is exposed to self-antigen. If a B-cell is strongly self-reactive and binds with high affinity to a self-antigen, this cell is in most cases removed by clonal deletion (via apoptosis) [41]. A similar mechanism is also controlling the removal of autoreactive T cells in the thymus [42].

A very specific example of apoptosis during the maturation of immune cells was also described *by* Kerr, Wyllie and Currie. They noted ‘*the occurrence of […] tingible bodies in the germline centers of lymphoid follicles’*. They speculated that rapid cell proliferation in the germinal centre (GC) of the lymph node might cause this observation of apoptosis. We now know that B cells, which are activated by antigens and T cells, rapidly proliferate and acquire somatic hypermutations of the immunoglobulin V regions, to improve the affinity of the B-cell receptor (BCR) to the antigen. The majority of clones, however, will have decreased affinity to the antigen and therefore need to be removed [43]. These defective B-cell clones die via apoptosis because they lose the interaction with the antigen and T cells, thereby losing pro-survival signals. They are quickly cleared by macrophages (called tingible body macrophages), that are a characteristically histological feature of germinal centres [44].

## Apoptosis in cancer

The transformation of a normal to a malignant cell is a multistep process, resulting from genome instability and/or inflammation. During the development of cancer, the transforming cells have to reprogramme their biological processes to evade natural defence mechanisms against tumour development. Inhibition of apoptosis is one of the mechanisms in the transformation of cells that promotes cancer development and progression [45]. The apoptotic programme can be triggered by several physiological stresses that indicate the initiation of a transformative process. DNA damage, oncogenic signalling and hyper-proliferation can all serve as a signal for cells to induce apoptosis [46]. One central player in activating apoptosis is the tumour suppressor p53. DNA breaks and other chromosomal abnormalities lead to p53 activation, which in turn stimulates apoptosis in a transcription-dependent and transcription-independent manner. Active p53 transcribes the pro-apoptotic proteins PUMA, NOXA and BAX to induce apoptosis [47]. In addition, cytoplasmic p53 can also bind to anti-apoptotic BCL-2 and BCL-xL to abolish the inhibition of BAX/BAK [48]. Furthermore, elevated oncogenic signalling by MYC can also lead to apoptosis (partly through BIM) [49].

Therefore resisting cell death programmes is a central strategy of cancer cells to manifest transformation and to maintain tumour growth [45]. In a wide variety of human cancers, the disequilibrium between pro- and anti-apoptotic BCL-2 family proteins promotes cancer cell survival. Inhibition or downregulation of pro-apoptotic proteins is widely observed. In addition, elevated levels of anti-apoptotic BCL-2 proteins are also an effective strategy used by cancer cells to evade programmed cell death [50]. There is much evidence that Bcl-2 proteins are also regulated by miRNAs. Increased expression of these biomolecules can silence mRNA and translation of pro-apoptotic proteins [51]. In addition, the expression levels of c-FLIP [52], IAPs [53] and caspases [54] are also altered in human cancer cells leading to reduction of apoptosis and promotion of carcinogenesis.

Kerr, Wyllie and Currie already noted that induction of apoptosis could be an avenue to treat cancer. As already mentioned above, they observed *‘an increase in apoptosis after irradiation’* in ‘*squamous cell carcinomata’* [5]. While radiotherapy remains a potent anticancer therapy, modern anticancer drugs also try to target and restore dysregulated apoptotic pathways, to selectively eliminate cancer cells [55]. For this goal, different therapeutic approaches have been used to target components of the intrinsic or extrinsic apoptotic pathway or by restoring the activity of tumour suppressors. BH3-mimetics are a class of promising small molecules that target anti-apoptotic BCL-2 proteins. Venetoclax, a selective BCL-2 inhibitor, has already been approved in the clinic for the treatment of patients with CLL or AML [56]. Other clinical trials are using compounds targeting IAPs (with SMAC-mimetics [57]) or death receptors [58]. In addition, drugs that target MDM2 [59], the inhibitor of p53, or molecules that restore the wild-type activity of mutant p53 [60] are also under clinical investigation.

Paradoxically, Kerr, Wyllie and Currie observed that *‘spontaneous and continuous death of cells is an inherent property of malignant neoplasms’*. This observation challenges the dogma that resisting cell death is a hallmark of cancer, but it has been shown more recently that apoptosis can also act as a tumour–promoting mechanism [45, 61, 62]. Conceptually, apoptosis in healthy tissues can drive clonal selection of premalignant cells and the subsequent outgrowth of more aggressive subclones [63]. This model has been proven experimentally by deleting the pro-apoptotic BH3-only protein PUMA, which abolishes tumour formation in a thymic T-cell lymphoma model [62]. Furthermore, ablation of the anti-apoptotic protein MCL-1 in hepatocytes induces widespread induction of apoptosis in the liver and results in the spontaneous development of hepatocellular carcinoma (HCC). This phenotype can be reversed by co-deleting Bak [64]. In addition, high levels of apoptosis have been observed in aggressive tumours of multiple cancer types. The quick and efficient removal of apoptotic bodies ensures that apoptosis causes *‘no inflammation’* as already noted by Kerr, Wyllie and Currie. Apoptosis is indeed considered to be one of the programmed cell death mechanisms that is immunologically silent. Nevertheless, dying cells are not an inert object and in addition to find-me signals, they can release a variety of factors that influence the survival and proliferation of surrounding cells. Prostaglandin E_2,_ for example, is produced in dying cells by a caspase-3-dependent mechanism and can stimulate the proliferation of cancer stem cells [65]. In addition, the phagocytosis of apoptotic bodies by tumour-associated macrophages (TAMs) can cause the release of several factors that promote proliferation, angiogenesis and cancer progression [66]. This dual role of apoptosis in cancer could potentially explain the fact that cancer therapies that target the apoptotic machinery often fail, resulting in tumour relapse.

**Fig. 2:**
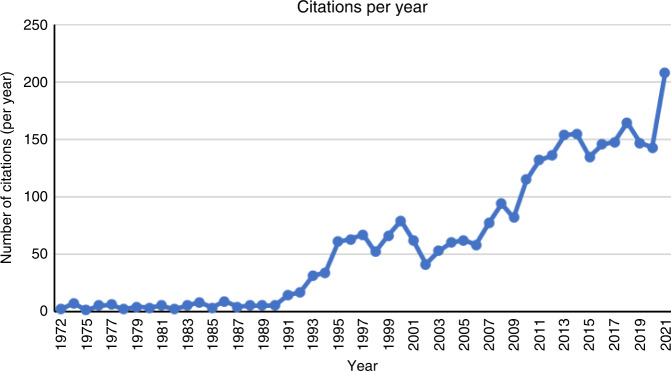
Citations per year in the period 1972–2021 of ref. [2]. Citations collated from NCBI.

In summary, it is undoubtedly clear that this publication in the *British Journal of Cance*r was a landmark study as judged by its enduring and increasing citations, with over 20,000 citations to date (Fig. 2). Moreover, not only was the study the first to describe apoptosis, but the subsequent study of apoptosis has led to the identification of several additional other forms of programmed cell death [3]. Interestingly, while some of these forms of cell death are mechanistically distinct, others involve key molecules that also have a role in apoptosis. For example, inducers of extrinsic apoptosis under certain conditions can trigger necroptosis, whereas pyroptosis is controlled by pro-inflammatory caspases. Moreover, caspase-8, the proximal caspase involved in most forms of extrinsic cell death also act as a repressor of necroptosis, indicating connections and interplay between different modes of cell death [67]. For example, it can be the case that analysis of molecular components of cell death pathways is not always sufficient to discern the involvement of one form of cell death versus another, and analysis of morphological characteristics are therefore also required. As such, Kerr, Wyllie and Currie is not only just a historical landmark publication in our understanding of cell viability, but it is also very much alive in current studies focused on the analysis of apoptosis in both normal physiology and multiple forms of disease.
